# Kidney volume and function of low-birth-weight children at 5 years: impact of singleton and twin birth

**DOI:** 10.1007/s00467-024-06554-8

**Published:** 2024-10-25

**Authors:** Patrik Konopásek, Aneta Kodytková, Peter Korček, Monika Pecková, Martina Frantová, Martin Kočí, Eva Flachsová, Karel Kotaška, Zbyněk Straňák, Jan Janda, Jakub Zieg

**Affiliations:** 1 Department of Pediatrics, Second Faculty of Medicine, Charles University and Motol University Hospital, V Úvalu 84, Praha 5, Prague, 15006 Czech Republic; 2 Institute for the Care of Mother and Child, Neonatal Intensive Care Unit, Prague, Czech Republic; 3 Institute of Applied Mathematics and Information Technologies, Faculty of Science, Charles University, Prague, Czech Republic; 4 Department of Obstetrics and Gynecology, Second Faculty of Medicine, Neonatal Unit, Charles University and Motol University Hospital, Prague, Czech Republic; 5 Department of Radiology, Second Faculty of Medicine, Charles University and Motol University Hospital, Prague, Czech Republic; 6 Department of Medical Chemistry and Clinical Biochemistry, Second Faculty of Medicine, Charles University and Motol University Hospital, Prague, Czech Republic; 7 Third Faculty of Medicine, Charles University, Prague, Czech Republic

**Keywords:** 5-year-old children, Glomerular filtration rate, Kidney volume, Low birth weight, Prematurity, Twins

## Abstract

**Background:**

Many studies have demonstrated the association between low birth weight (LBW) and chronic kidney disease, estimated glomerular filtration rate (eGFR) and kidney volume (KV). However, studies on twins and those investigating numerous perinatal factors beyond LBW, and their associations with various kidney parameters are scarce.

**Methods:**

A two-center cross-sectional study on five-year-old LBW children was conducted between 2021 and 2023. 110 children were enrolled (8 LBW, 58 very LBW (VLBW), 44 extremely LBW (ELBW)); 56 were twins. We examined associations between birth weight (BW), various prenatal, perinatal and postnatal factors, and eGFR, KV, tubular abnormalities and kidney ultrasound abnormalities, both in singletons and twins.

**Results:**

In children with ELBW, eGFR correlated with BW (r = 0.55, *P* = 0.0018), while in those with BW ≥ 1000 g, eGFR remained constant. Other factors associated with decreased eGFR were hypertensive disorder of pregnancy (93.86 vs. 87.26 ml/min/1.73m^2^, *P* = 0.0285) in singletons, decreased growth velocity (β = 0.83, *P* = 0.0277) in twins, and lower total KV (tKV) and relative KV (rKV) in both singletons (r = 0.60, *P* < 0.0001 for tKV and r = 0.45, *P* = 0.0010 for rKV) and twins (β = 0.34, *P* < 0.0001 for tKV and β = 0.23, *P* = 0.0002 for rKV). Based on the multivariable models excluding KV, BW and gestational age were associated with eGFR in singletons, while male gender, BW, growth velocity, and coffee drinking during pregnancy were associated with eGFR in twins. However, in models that included KV, BW, gestational age and growth velocity were no longer significant. Total KV was associated with BW (r = 0.39, *P* = 0.0050 for singletons; β = 2.85, *P* < 0.0001 for twins), body mass index (r = 0.34, *P* = 0.0145 for singletons; β = 8.44, *P* < 0.0001 for twins), and growth velocity (β = 1.43, *P* = 0.0078). Twins born small for gestational age had lower tKV (70.88 vs 89.20 ml, *P* < 0.0001). Relative KV showed similar associations. Relative kidney volumes were significantly lower for both kidneys compared to the reference population (55.02 vs 65.42 ml/m^2^,* P* < 0.0001 for right kidney and 61.12 vs 66.25 ml/m^2^, *P* = 0.0015 for left kidney); however, only 8.6% of children had rKV below 10^th^ percentile.

**Conclusion:**

Many factors affect eGFR and KV, some of them differ between twins and singletons. Based on multivariable models, eGFR seems to be better predicted by KV than by BW and gestational age in LBW children. Relative kidney volumes were significantly lower in our cohort compared to the reference population, but only 8.6% of rKV were below 10^th^ percentile.

**Graphical Abstract:**

**Figure:**
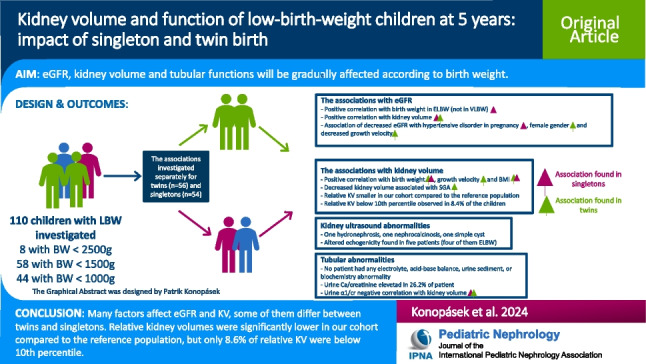
A higher resolution version of the Graphical abstract is available as Supplementary information

**Supplementary Information:**

The online version contains supplementary material available at 10.1007/s00467-024-06554-8.

## Introduction

Low birth weight (LBW), defined as a birth weight less than 2500 g, is a global concern, with an estimated prevalence of 15–20% of all births worldwide. It is associated with significant perinatal and postnatal morbidity and mortality [1]. Some factors, such as maternal anemia or low calcium intake, can be effectively addressed, potentially preventing LBW in many cases. Implementing measures to prevent these factors can significantly reduce the incidence of LBW and contribute to public health [1].

LBW has been found to be associated with chronic kidney disease (CKD). A meta-analysis in 2007 reported that there is a 70% greater risk of developing CKD in individuals born with LBW [2]. Normal kidney development is affected in premature/LBW individuals. These children are born with fewer nephrons and their total nephron count correlates positively with birth weight (BW). A decreased nephron count leads to hyperfiltration of residual nephrons with subsequent glomerular hypertrophy, which also correlates with BW [3, 4]. Persistent hyperfiltration leading to glomerular injury and subsequent glomerular sclerosis is believed to be the main mechanism of CKD development in LBW individuals [5]. Nephrogenesis may continue in some preterm infants after birth. However, their development may not reach its full potential and is associated with growth of abnormal glomeruli. Such kidneys are likely more easily damaged by acute kidney injury (AKI) and other injurious factors [5–7]. This “second hit” is commonly seen in preterm/LBW children and is believed to contribute to CKD in LBW individuals together with the hyperfiltration–glomerulosclerosis theory [5]. Premature children have higher prevalence of AKI, the risk of which increases with decreasing BW [5]. AKI is a known risk factor for CKD, which has also been demonstrated in the pediatric population [8].

Many studies demonstrated higher risk for CKD in LBW children and adolescents. Nonetheless, most of the studies focused on a few specific risk factors and outcomes and mainly compared LBW children, particularly those born with extremely low birth weight (ELBW), with individuals born with normal birth weight [9–19].

The purpose of this study was to evaluate the associations between BW, various prenatal, perinatal and postnatal factors, and kidney function, kidney volume (KV) and tubular and kidney ultrasound abnormalities in a cohort of 5-year-old children born with LBW, with a particular focus on very low birth weight (VLBW) and ELBW patients. Our aim was to offer a comprehensive understanding of this population and explore various factors potentially associated with different kidney parameters in both singletons and twins. Our secondary objective was to investigate the association between KV and other kidney outcomes, examining it both independently and in combination with the prenatal, perinatal, and postnatal factors.

## Patients and methods

This was a two-center cross-sectional study conducted in Prague, Czech Republic (*Institute for the Care of Mother* and *Motol University Hospital*) between 2021 and 2023. The goal of the study was to evaluate the associations between BW, various prenatal, perinatal and postnatal factors, and kidney function, KV and kidney ultrasound abnormalities and the prevalence of tubular abnormalities in children at 5 years of age who had been born with LBW, focusing on those born with VLBW and ELBW. We selected the age of 5 years for early screening of kidney abnormalities associated with LBW, as younger children may be less likely to exhibit such signs. Moreover, many perinatal centers in the Czech Republic provide follow-up care for LBW children until 5 years of age and older children may no longer be under close follow-up. All VLBW and ELBW children aged 5 years born from 01/01/2016 to 31/09/2017 in one of the centers, without any known pathogenic variant associated with kidney disease and living within a 1-h travel radius of Prague, were contacted and offered participation in the study. In the case of twins, both were automatically offered to be involved in the study even if BW for one of them was ≥ 1500 g to allow analysis of twins. LBW was defined as BW < 2500 g, VLBW as BW < 1500 g, ELBW as BW < 1000 g and small for gestational age (SGA) as BW < 2 standard deviation scores (SDS) below the mean [20].

Growth and weight parameters measured by their primary care provider during periodic follow-up over the first year of life were collected from each patient’s personal charts. Prenatal and perinatal data, which included BW, gestational age, type of birth, use of antenatal corticosteroids for fetal lung maturation (both complete and incomplete treatments), any hypertensive disorder of pregnancy, bronchopulmonary dysplasia, patent ductus arteriosus, any occurrence of neonatal sepsis from birth to discharge, any single dose of furosemide and aminoglycosides, any single dose of nonsteroidal analgesics (NSAID), necrotizing enterocolitis and neonatal AKI were collected from the obstetrics and neonatology electronic and printed medical records. During the entire newborn hospitalization period, AKI was defined as elevation of serum creatinine and/or oliguria based on the modified KDIGO neonatal AKI classification [21]. Additional data, which included family history of hypertension (HT) (presence of maternal and/or paternal HT), coffee drinking during pregnancy (at least one cup of caffeinated coffee daily), gestational diabetes, smoking and alcohol use during pregnancy and maternal anemia were collected during an interview with the parents.

The anthropometry was performed by a single experienced anthropologist at 5 years of age. The height was obtained via wall mounted Seca stadiometer (A-226 manufactured by Trystom in Olomouc, Czech Republic) with an accuracy of 1 mm, and weight was based on values measured on the calibrated weighing electronic scale with an accuracy of 0.1 kg (TH200, manufactured by Tonava in Upice, Czech Republic). The body mass index (BMI) was calculated using the standard formula as weight (in kg) divided by height (in meters) squared. Height and BMI SDS were generated from the RustCZ software using the learning management system LMS method based on the 6th Czech Nationwide Anthropological Survey of Children and Adolescents [22, 23]. Growth velocity (cm/year) from birth to the first year of life was calculated from the first measured body length and the body length measured at the time of the first birthday. The evaluated period did not exceed 12 months.

The blood samples in our cohort of 5-year-old LBW children were obtained after 12 h of fasting and urine samples were collected from a first morning urine collection. The samples were analyzed immediately after being transported to the laboratory. For the analysis of serum and urine, the Atellica Solutions CH 930 analyzer (Siemens, USA) was used for measurement of Na, K, Cl, P, Ca, Mg, urea, creatinine, cystatin C (CyC), α1-microglobulin and β2-microglobulin. The urine sediment was analyzed using the Atellica 1500, which combines the chemical module Clinitek Novus analyzer and the sediment module Atellica Automated Urinalysis System 800 (Siemens, USA). Blood gasses and acid–base parameters were assayed on RapidLab 1265, (Siemens, USA). The local laboratory reference values used for comparison have been published [24]. Estimated glomerular filtration rate (eGFR) was calculated using the creatinine-CyC-based chronic kidney disease in children cohort study (CKiD) equation from 2012 [25]. A urine calcium/creatinine ratio (U-ca/cr) > 0.65 mmol/mmol was considered to represent hypercalciuria. The urine α1-microglobulin/creatinine ratio (U-α1/cr) and β2-microglobulin/creatinine ratios (U-β2/cr) were evaluated as continuous variables.

Kidney ultrasound was performed at 5 years of age by two experienced physicians using the Canon diagnostic ultrasound system model Toshiba Xario 100 with Toshiba convex array transducer model PVU-674BT with a frequency range of 3.6–9.2 MHz. The examinations were performed with the subjects lying in a prone position. For each child, kidney length, width and thickness in cm were calculated as the average of three measurements. KV in ml for each kidney was calculated using the formula: (kidney length × kidney width × kidney thickness) × Pi/6 and total KV (tKV) as a sum of both kidneys’ KV, and relative kidney volume (rKV) in ml/m^2^ was established by dividing tKV by BSA according to previous study [9].

**Fig. 1:**
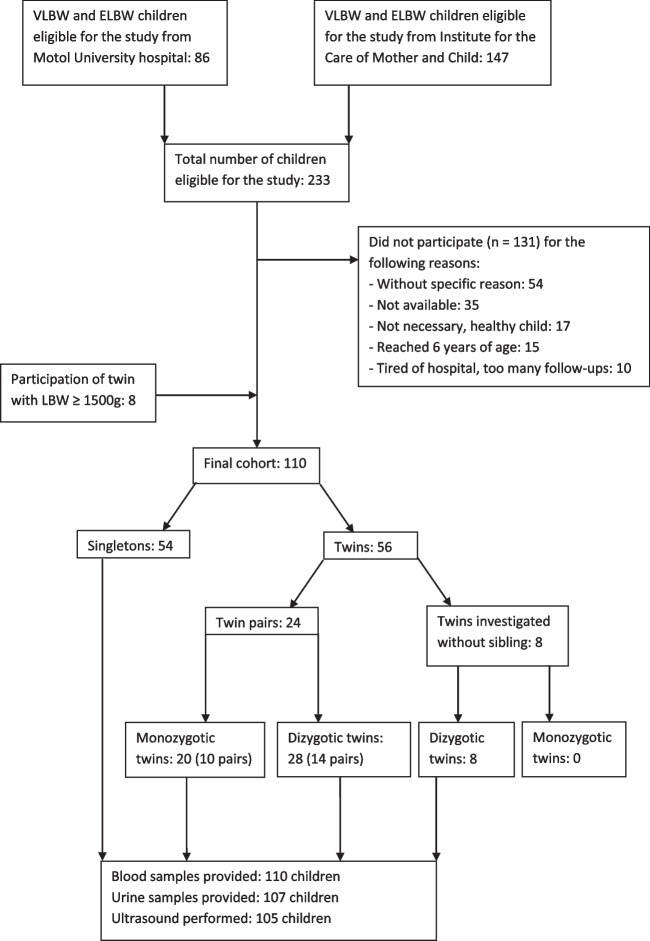
Flowchart. ELBW–Extremely low birth weight, LBW–Low birth weight, VLBW–Very low birth weight

In total, 233 children were eligible for the study, 110 were enrolled (with inclusion of eight LBW twin siblings with BW ≥ 1500 g). Fifty-six were twins (eight of whom were investigated without their siblings, and 48 were paired twins) and 54 were singletons. All children had blood samples taken; all but three were able to provide first morning urine. Five children refused kidney ultrasound examination (Fig. 1).

Statistical analysis was performed in the statistical package R, version 3.6.1. A* P*-value ≤ 0.05 was considered statistically significant. Analyses of the associations were performed separately in twins and singletons because the twins consist of dependent data, and they had different distributions of investigated parameters in our cohort (Table 1). Associations with eGFR, tKV, rKV and tubular markers were tested for birth weight, gestational age, BMI, growth velocity, SGA, sex, birth weight < 1000 g, family history of HT, type of birth, antenatal corticosteroids use, coffee drinking during pregnancy, hypertensive disorders of pregnancy, maternal anemia, bronchopulmonary dysplasia, neonatal sepsis, use of furosemide and aminoglycosides, patent ductus arteriosus, use of NSAID (in twins) and neonatal AKI (in singletons). Gestational diabetes, smoking and alcohol use during pregnancy, and necrotizing enterocolitis were excluded because of the insignificant numbers of pregnancies with these factors. While investigating associations between the described factors and KV, we also included KV as an exposure/predictor in the analysis of other kidney outcomes, examining it both independently and in combination with the prenatal, perinatal, and postnatal factors to assess any potential associations.

**Table 1:** Cohort demographic characteristics presented as mean ± standard deviations or total number + percentage

*Parameter*	*Cohort (n* = *110)*	*Singletons (n* = *54)*	*Twins (n* = *56)*
Age at examination (years)	5.61 ± 0.20	5.62 ± 0.18	5.60 ± 0.22
Male gender	53 (48.18%)	31 (57.41%)	22 (39.29%)
Birth weight (g)	1109.7 ± 315.4	1003.5 ± 271.4	1212.0 ± 323.1
Gestational age (weeks)	29.0 ± 2.8	28.0 ± 2.8	29.9 ± 2.5
Small for gestational age	17 (15.5%)	8 (14.81%)	9 (16.1%)
Body mass index (standard deviation score)	–0.86 ± 1.09	–0.74 ± 1.13	–0.96 ± 1.04
Body surface area (m^2^)	0.74 ± 0.07	0.75 ± 0.08	0.74 ± 0.07
Growth velocity (cm/year)	30.86 ± 3.59	30.86 ± 3.39	30.87 ± 3.79

Absolute numbers with percentage and mean values with standard deviation (SD), and lower and upper limits were used for descriptive statistics. In the singletons group, we used Welch t-tests for testing associations with categorical variables and correlation coefficients and regression models for continuous variables and multivariate models. In the twin’s group we used regression models with generalized estimating equations to adjust for dependencies between twins. Comparison between dominant and non-dominant twins were conducted using a paired t-test. The right and left rKVs from the whole cohort were compared to the previously published data using a two tailed t-test [26].

## Results

### Complete cohort

In our cohort, eight (7.3%) children were born with a LBW (BW < 2500 g), 58 (52.7%) with a VLBW (BW < 1500 g) and 44 (40%) with an ELBW (BW < 1000 g). Fifty-seven (51.8%) were female and 53 (48.2%) were male. The mean BW was 1109.7 ± 315.4 (370–1890) g and the mean gestational age was 29.0 ± 2.8 (23–34) weeks. All children were of White/Caucasian ethnicity. Fifty-six patients were born as twins; 24 pairs were investigated together, while 8 children were investigated without their siblings. Characteristics of the cohort may be found in Table 1, Table 2, and Supplementary Material 2.

**Table 2:** Kidney volume and eGFR in the cohort presented as mean ± standard deviations or total number + percentage or SDS relative to the reference population [26]

*Parameter*	*Cohort (n* = *110)*	*Singletons (n* = *54)*	*Twins (n* = *56)*
eGFR (ml/min/1.73 m^2^)	91.58 ± 10.83	91.42 ± 11.18	91.73 ± 10.58
eGFR < 90 ml/min/1.73 m^2^	49 (44.55%)	28 (51.85%)	21 (37.50%)
Right kidney volume (ml)	41.18 ± 8.49	42.44 ± 8.23	39.98 ± 8.64
Left kidney volume (ml)	45.75 ± 9.34	44.95 ± 7.95	46.51 ± 10.51
Total kidney volume (ml)	86.93 ± 16.37	87.40 ± 15.01	86.49 ± 17.69
Right kidney relative volume (ml/m^2^)	55.02 ± 9.03	56.38 ± 8.96	53.73 ± 8.99
Right kidney relative volume SDS relative to the reference population [26]	–0.68	–0.60	–0.78
Right kidney relative volume below 10th percentile compared to reference population [26]	11 (10.5%)	4 (7.8%)	7 (13.0%)
Left kidney relative volume (ml/m^2^)	61.12 ± 9.89	59.81 ± 8.99	62.36 ± 10.61
Left kidney relative volume SDS relative to the reference population [26]	–0.32	–0.40	–0.24
Left kidney relative volume below 10th percentile compared to reference population [26]	4 (3.8%)	2 (3.9%)	2 (3.7%)
Total kidney relative volume (ml/m^2^)	123.95 ± 24.95	125.23 ± 26.91	122 ± 23.14
Total kidney relative volume SDS relative to the reference population [26]	–0.26	–0.22	–0.32
Total kidney relative volume below 10th percentile compared to reference population [26]	9 (8.6%)	4 (7.8%)	5 (9.3%)

*eGFR* – Estimated glomerular filtration rate, *SDS* – Standard deviation score

The mean BW in those who were eligible but did not participate in the study was 1105.1 ± 260 g, gestational age 29.2 ± 2.7 weeks, and 39.7% were born with ELBW. Differences of BW and gestational age between both groups were not significant (*P* = 0.9397 and *P* = 0.4864, respectively).

### The associations with glomerular filtration rates

**Fig. 2:**
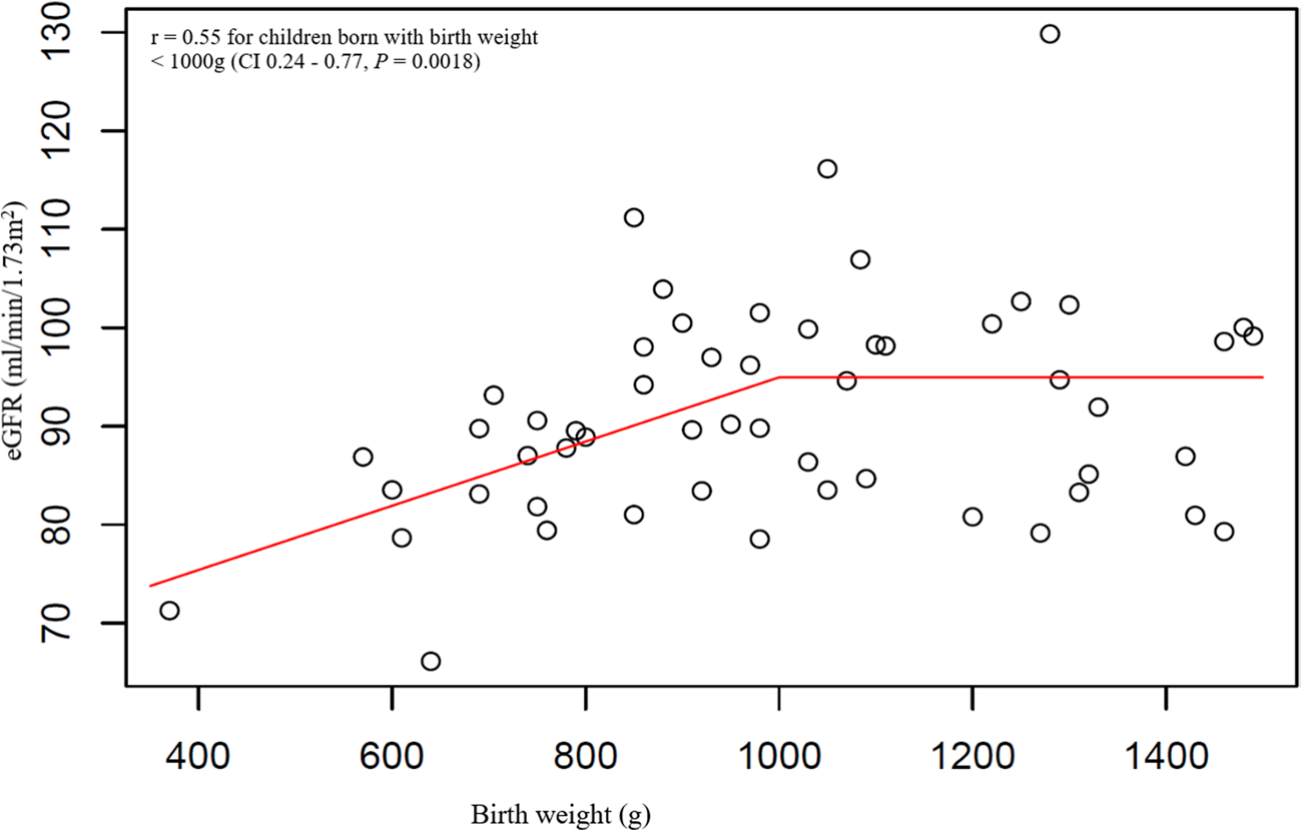
The correlation between birth weight and estimated glomerular filtration rate. eGFR–Estimated glomerular filtration rate, g – Grams, min–Minute

In singletons, eGFR was positively correlated with BW, tKV and rKV (Table 3). Based on the linear models for eGFR, the association between BW and eGFR exists only for BW < 1000 g, the association is of linear shape with function described as y = 62.37 + 0.0326x (r = 0.55, 95% CI 0.24–0.77, *P* = 0.0018). BW > 1000 did not influence eGFR (Fig. 2). From the categorical variables, only hypertensive disorders of pregnancy showed an association with lower eGFR (eGFR 93.86 vs. 87.26 ml/min/1.73 m^2^, 95% CI 0.73–12.48, *P* = 0.0285) (Supplementary Material 3). The multivariable model that did not include either tKV or rKV showed that only BW and gestational age were associated with eGFR. In two children with the same gestational age, an increase in BW by 100 g led to an increase in eGFR by 3 ml/min/1.73 m^2^. In two children with the same BW, an increase in gestational age by 1 week led to a decrease in eGFR by 1.964 ml/min/1.73 m^2^. The multivariable model that included tKV showed that only tKV and AKI were associated with eGFR. When controlling for the other parameter, children with AKI had an eGFR 10.34 ml/min/1.73 m^2^ lower than non-AKI children. An increase in tKV by 1 ml led to an increase in eGFR by 0.429 ml/min/1.73 m^2^. The multivariable model with rKV instead of tKV showed similar results (Table 4).

**Table 3:** Correlations and regression models for associations between continuous variables and eGFR, kidney volume and relative kidney volume

Correlations between estimated glomerular filtration rate and continuous variables in singletons			
Investigated parameter	Correlation coefficient	95% CI	*P-*Value
* Birth weight*	*0.31*	*0.05*–*0.53*	0.0206*
Gestational age	0.08	-0.20–0.34	0.5828
* Total kidney volume*	*0.60*	*0.38*–*0.74*	< 0.0001*
* Relative kidney volume*	*0.45*	*0.19*–*0.64*	0.0010*
Body mass index – standard deviation score	0.13	-0.14–0.39	0.3402
Growth velocity	0.23	-0.05–0.47	0.1028
Regression models for associations between estimated glomerular filtration rate and continuous variables in twins			
Investigated parameter	Regression coefficient	95% CI	*P-*Value
Birth weight	0.76 for each 100 g	-0.32–1.84	0.1697
Gestational age	-0.52 for each 1 week	-2.23–1.19	0.5488
* Total kidney volume*	*0.34 for each 1 ml*	*0.21*–*0.46*	< 0.0001*
* Relative kidney volume*	*0.23 for each 1 ml/m*^*2*^	*0.11*–*0.35*	0.0002*
Body mass index – standard deviation score	1.26 for 1 standard deviation score	-1.83–4.34	0.4243
* Growth velocity*	*0.83 for each 1 cm/year*	*0.09*–*1.56*	0.0277*
Correlations between total kidney volume and continuous variables in singletons			
Investigated parameter	Correlation coefficient	95% CI	*P-*Value
* Birth weight*	*0.39*	*0.13–0.60*	0.0050*
Gestational age	0.12	-016–0.38	0.4016
* Body mass index – standard deviation score*	*0.34*	*0.07–0.56*	0.0145*
Growth velocity	0.24	-0.05–0.48	0.0991
Correlations between relative kidney volume and continuous variables in singletons			
Investigated parameter	Correlation coefficient	95% CI	*P-*Value
Birth weight	0.19	-0.09–0.44	0.1869
Gestational age	0.03	-0.25–0.31	0.8195
Body mass index – standard deviation score	-0.10	-0.36–0.18	0.4902
Growth velocity	0.12	-0.17–0.38	0.4179
Regression models for associations between total kidney volume and continuous variables in twins			
Investigated parameter	Regression coefficient	95% CI	*P-*Value
* Birth weight*	*2.85 for each 100 g*	*1.808–3.885*	< 0.0001*
Gestational age	-1.50 for each 1 week	-3.501–0.511	0.1440
* Body mass index – standard deviation score*	*8.44 for 1 standard deviation score*	*4.497–12.373*	< 0.0001*
* Growth velocity*	*1.43 for each 1 cm/year*	*0.376–2.487*	0.0078*
Regression models for associations between relative kidney volume and continuous variables in twins			
Investigated parameter	Regression coefficient	95% CI	*P-*Value
* Birth weight*	*2.89 for each 100 g*	*1.436–4.343*	0.0001*
Gestational age	0.20 for each 1 week	-2.597–2.990	0.8905
* Body mass index – standard deviation score*	*7.22 for 1 standard deviation score*	*1.358–13.090*	0.0158*
Growth velocity	0.66 for each 1 cm/year	-0.529–1.839	0.2783

*eGFR* – Estimated glomerular filtration rate

**Table 4:** Multivariable models

Singletons, multivariable model for eGFR without kidney volume in the model		
Parameter	Regression coefficient	*P-*Value
Intercept	11.901	< 0.0001
Birth weight	0.030	0.0014
Gestational age	− 1.964	0.0227
Singletnos, multivariable model for eGFR with total kidney volume in the model		
Parameter	Regression coefficient	*P-*Value
Intercept	44.66	< 0.0001
Total kidney volume	0.429	< 0.0001
Acute kidney injury	− 10.34	0.0335
Singletnos, multivariable model for eGFR with relative kidney volume in the model		
Parameter	Regression coefficient	*P-*Value
Intercept	61.05	< 0.0001
Relative kidney volume	0.165	0.0035
Acute kidney injury	− 11.05	0.0387
‍Twins, multivariable model for eGFR without kidney volume in the model		
Parameter	Regression coefficient	*P-*Value
‍ Intercept	57.169	< 0.0001
‍ Birth weight	0.013	0.0092
‍ Male gender	7.373	0.0246
‍ Growth velocity	0.761	0.0181
‍ Coffee drinking during pregnancy	9.326	0.0092
Twins, multivariable model for eGFR with total kidney volume in the model		
Parameter	Regression coefficient	*P-*Value
Intercept	70.767	< 0.0001
Male gender	5.940	0.0389
Total kidney volume	0.273	< 0.0001
Coffee drinking during pregnancy	5.974	0.0116
Twins, multivariable model for eGFR with relative kidney volume in the model		
Parameter	Regression coefficient	*P-*Value
Intercept	73.290	< 0.0001
Male gender	6.068	0.0499
Relative kidney volume	0.179	0.0006
Coffee drinking during pregnancy	7.269	0.0023
Singletons, multivariable model for total kidney volume		
Parameter	Regression coefficient	*P-*Value
Intercept	126.707	< 0.0001
Birth weight	0.047	0.0002
Gestational age	− 3.097	0.0096
Twins, multivariable model for total kidney volume		
Parameter	Regression coefficient	*P-*Value
Intercept	168.751	< 0.0001
Birth weight	0.025	< 0.0001
Gestational age	− 3.589	< 0.0001
Body mass index	6.171	0.0086
Twins, multivariable model for relative kidney volume		
Parameter	Regression coefficient	*P-*Value
Intercept	86.431	< 0.001
Birth weight	0.020	0.0298
Small for gestational age	− 12.855	0.0147

In twins, eGFR was positively correlated with growth velocity, tKV and rKV. BW was not correlated with eGFR in twins, probably due to the low number of twins born with ELBW (Table 3). Coffee drinking during pregnancy was the only categorical variable associated with eGFR, the effect was protective (eGFR 89.21 vs. 100.94 ml/min/1.73 m^2^, 95% CI -16.55 to -7.43, *P* < 0.0001) (Supplementary Material 3). The multivariable model that did not include either tKV or rKV showed that male gender, higher BW, higher growth velocity, and coffee consumption during pregnancy were associated with higher eGFR. Specifically, for the same values of the other parameters, males had eGFR that was 7.373 ml/min/1.73 m^2^ higher, each 100 g of BW increased eGFR by 1.30 ml/min/1.73 m^2^, each 1 cm/year of growth velocity increased eGFR by 0.761 ml/min/1.73 m^2^, and offspring of coffee drinking mothers had an eGFR that was 9.326 ml/min/1.73 m^2^ higher. The multivariable model that included tKV showed that, when controlling for other parameters, male sex (eGFR 5.94 ml/min/1.73 m^2^ higher), tKV (each 1 ml increase in tKV associated with an eGFR increase of 0.237 ml/min/1.73 m^2^) and coffee drinking during pregnancy (eGFR 5.974 ml/min/1.73 m^2^ higher) were associated with better eGFR. Similar results were shown by the multivariable model with rKV instead of tKV (Table 4).

In the analysis of twin pairs, eGFR did not significantly differ between dominant and non-dominant twins (difference 2.48 ml/min/1.73 m^2^, 95% CI -1.95–6.70, *P* = 0.2587). The dominant twins had a 0.4 higher BMI SDS than the non-dominant twins. The difference nearly reached statistical significance (95% CI -0.007–0.807,* P* = 0.0536). Growth velocity did not significantly differ between them (95% CI -1.496–1.279,* P* = 0.8731).

### The associations with kidney volume

**Fig. 3:**
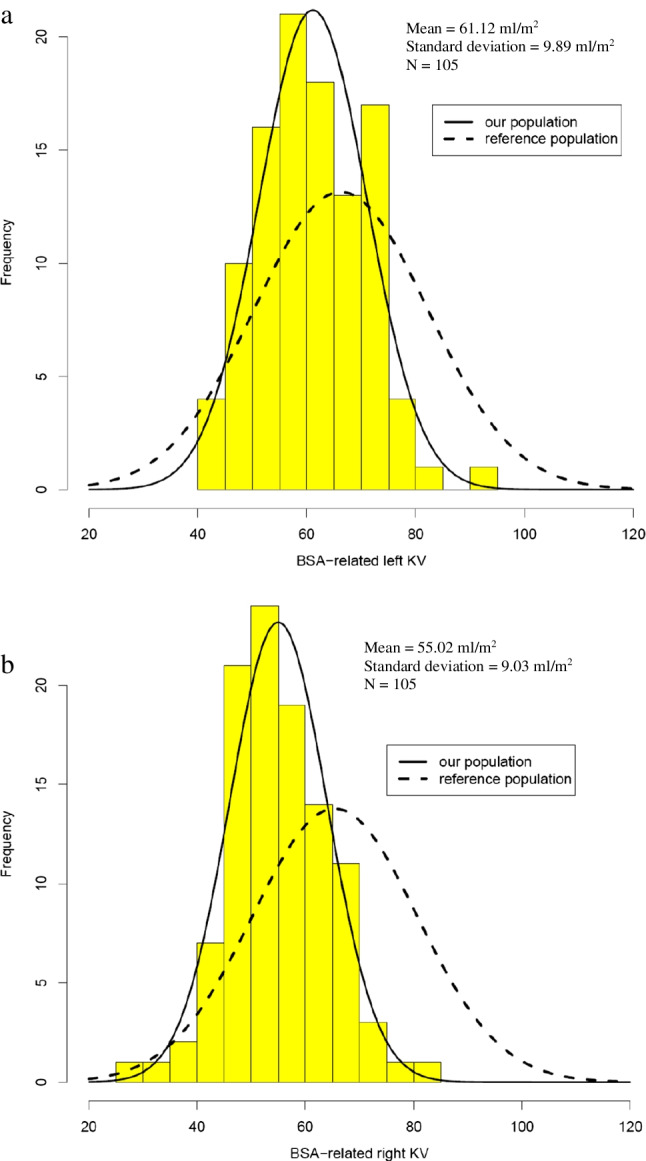
Distribution of relative kidney volumes for both kidneys, superimposed with reference population data adjusted to match cohort size, a – Left kidney, b – Right kidney. BSA–Body surface area, KV-Kidney volume

Both kidneys had a normal distribution of rKV (Fig. 3). The mean relative volume of the left kidney was significantly higher than that of the right kidney (61.12 vs. 55.02 ml/m^2^, 95% CI 4.324–7.885, *P* < 0.0001). Compared to previously reported values of Scholbach et al. [26], both the right (55.02 ± 9.03 vs. 65.42 ± 15.20 ml/m^2^,* P* < 0.0001) and left (61.12 ± 9.89 vs. 66.25 ± 15.93 ml/m^2^, *P* = 0.0015) kidneys had significantly lower mean rKV. However, only 8.6% of children had rKV below 10^th^ percentile (Table 2, Fig. 3).

In singletons, tKV was positively correlated with BW and BMI. From the categorical variables, only BW < 1000 g was associated with a lower tKV (92.54 vs. 82.83 ml, 95% CI 1.64–17.80, *P* = 0.0194). Interestingly, no association was found with rKV (Table 3, Supplementary material 3). The multivariable model showed that only BW and gestational age were associated with tKV. In two children with the same gestational age, an increase in BW by 100 g increased tKV by 4.7 ml, and in two children with the same BW, an increase in gestational age by 1 week decreased tKV by 3.097 ml (Table 4).

In twins, tKV was positively correlated with BW, BMI and growth velocity. For rKV, the association remained with BW and BMI (Table 3). From categorical variables, being born SGA was associated with both lower tKV (89.20 vs. 70.88 ml, 95% CI 11.78–22.89, *P* < 0.0001), and rKV (125.23 vs. 108.47 ml/m^2^, 95% CI 12.30–25.82, *P* < 0.0001) and coffee drinking during pregnancy with both higher tKV (82.69 vs. 99.76 ml, 95% CI -31.01 to -4.53, *P* = 0.0085), and rKV (118.61 vs. 137.24 ml/m^2^, 95% CI -36.00 to -3.84, *P* = 0.0152) (Supplementary Material 3). The multivariable model showed that BW, gestational age and BMI were associated with tKV. Specifically, in two children with the same BMI and gestational age, for each 100 g increase in BW, tKV increased by 2.5 ml; for two children with the same BW and BMI, each 1 week decrease in gestational age was associated with a 3.589 ml increase in tKV; and in two children with the same BW and gestational age, each 1 SDS increase in BMI was associated with a 6.171 ml increase in tKV. The same model for rKV showed the association with BW (an increase in BW by 100 g increased rKV by 2 ml/m^2^) and SGA (rKV 12.855 ml/m^2^ lower in SGA born children) (Table 4).

In the analysis of twin pairs, the dominant twins had a higher tKV than the non-dominant twins (difference 7.874 ml, 95% CI 1.772–13.997, *P* = 0.0137). Relative kidney volume did not significantly differ between dominant and non-dominant twins (difference 5.934 ml/m^2^, 95% CI -2.262–14.131, *P* = 0.1478).

### Kidney ultrasound abnormalities

There were only a few abnormalities on kidney ultrasound in our cohort. One patient had nephrocalcinosis, one had a simple cyst of 6 mm, and one had hydronephrosis. The most prevalent abnormalities were altered echogenicity mainly found in those with ELBW (one patient with hyperechogenic parenchyma, born at 600 g; three patients with altered corticomedullary differentiation, born with at 600, 610 and 1050 g; and one with both hyperechogenic parenchyma and altered corticomedullary differentiation, born at 370 g and presented as a case report [27]).

### Urine biochemistry, urinary sediment, and markers of tubular damage

No patients in our cohort had a significant abnormality in their urine biochemistry, urine sediment, serum electrolytes or acid–base balance suggesting significant tubulopathy. U-ca/cr was found to be elevated in 26.2% of patients. There was a negative correlation between U-α1/cr and both tKV and rKV in singletons (r =  − 0.44, CI − 0.64 to − 0.19,* P* = 0.0014 and r =  − 0.40, CI − 0.61 to − 0.14,* P* = 0.0041), small negative correlation with eGFR almost reached significance (r = –0.27, *P* = 0.0559). The same correlation was found in twins between U-α1/cr and rKV (regression coefficient − 0.006, CI − 0.0114 to − 0.0006,* P* = 0.0285). U-ca/cr was negatively associated with maternal coffee drinking (OR 4.76, 95% CI 1.32–17.15, *P* = 0.0171).

## Discussion

We report a comprehensive study exploring the associations between LBW, numerous prenatal, perinatal and postnatal factors, and various kidney parameters in 2 distinct populations: singletons and twins. It has been more than 30 years since Brenner et al. proposed the hypothesis that decreased glomerular count leads to hyperfiltration and glomerular hypertension due to decreased filtration surface area with subsequent glomerular sclerosis [2, 5, 28]. Numerous studies have aimed to investigate eGFR and KV in children and adolescents born with LBW [10–19]. In the study of Japanese adolescents, eGFR was found to be positively correlated with BW. LBW individuals and those who were overweight had higher odds for CKD stage 2 [10]. A few studies on 11-year-old children found significantly lower eGFR in ELBW individuals compared to the control group; in one study, those born SGA had even lower eGFR [11–13]. In two studies on young adolescents aged 12–15 years, those born with a LBW had lower eGFR than those born with normal BW, and eGFR was even lower in VLBW individuals [14, 15]. In a Polish longitudinal study, ELBW born children had significantly higher CyC and lower KV compared to the control group at age of 7 and 11 years [9]. Nine-year-old Bangladeshi LBW children were found to have lower eGFR compared to normal BW controls and lower KV at age 4.5 years than normal BW controls [16]. A study of 6–7-year-old children found a significantly lower KV in ELBW children compared to controls [17]. In the study of preschool children aged 5.7 ± 1.4 years, ELBW had significantly lower KV than VLBW children, but eGFR did not differ between the groups [18]. Young adults born at gestational age < 32 weeks were found to have significantly reduced KV, and renal structural anomalies were present in 8 of 51 individuals (nephrocalcinosis, pyelocaliceal dilatation, uretero-pelvic junction obstruction, ureter dilatation, extrarenal pelvis, and ureter duplication and ureterocele) [19].

In our study, we found a positive correlation between BW and eGFR in ELBW children, while in those born with BW ≥ 1000 g, eGFR remained constant. The explanation could be that in those born with BW ≥ 1000 g, the remaining nephrons are still able to maintain normal eGFR, while in ELBW children, the pool of nephrons is too small to maintain normal eGFR. KV showed positive correlation with eGFR in the whole cohort; the KV therefore seems to reflect indirectly the absolute pool of nephrons in 5-year-old children, but our results cannot be extrapolated to all populations. Based on the meta-analysis in adults from 2013, kidney size is not a suitable marker of nephron pool in adults [29]. We also found other factors associated with decreased eGFR, which were different in singletons (hypertensive disorders during pregnancy, AKI) and twins (decreased growth velocity, female gender). Based on the multivariable models excluding KV, BW and gestational age were associated with eGFR in singletons, while male gender, BW, growth velocity, and coffee drinking during pregnancy were associated with eGFR in twins. However, in models that included kidney volume, BW, gestational age, and growth velocity were no longer significant. These results indicate that KV appears to be a better predictor of eGFR than BW and gestational age at 5 years of age. In general, the results of our study support previous publications and show that many factors may have a significant impact on kidney health in LBW individuals. The association between LBW and CKD is very complex, with many variables that may influence the outcome.

The measurement of GFR has many significant limitations for routine practice; for that reason, an estimation using endogenous markers is the first-line examination. Creatinine is the most frequent marker used for GFR estimation. However, its use is limited by many non-GFR determinants. CyC seems to be less influenced by these determinants, but its use is limited by the price [30]. Designing a universal equation to precisely estimate GFR is one of the most challenging tasks in pediatric and adult nephrology. Many equations have been created for different populations, with combined creatinine and CyC equations showing the best performance [30]. We decided to use the creatinine-CyC-based CKiD equation; however, we found no study comparing different equations for eGFR with measured GFR in the LBW population. Therefore, we believe that the most appropriate equation for this specific population is unknown. This statement is supported by different equations used within the studies [9–18].

We describe a positive correlation between BW, BMI, growth velocity and KV. SGA was associated with lower KV in twins. Total KV was in general better associated with all the factors than rKV. Based on our multivariable analyses, both BW and BW relative to gestational age were associated with KV. Specifically, when comparing children with the same BW, those with a lower BW percentile for their gestational age had lower KV compared to those with lower gestational age but higher BW percentiles. The same pattern was observed for eGFR. Both BW and growth parameters were already demonstrated to correlate with KV, as previously mentioned [9, 16, 17, 31]. Relative kidney volumes of both kidneys in our cohort were significantly lower than those of the reference population reported by Scholbach et al. [26]. This result is not surprising, as many authors have found lower rKV in children and young adults with LBW compared to those with normal BW [9, 16, 19, 32]. However, only 10.5% of right rKVs, 3.8% of left rKVs, and 8.6% of the rKV for both kidneys in our study fell below 10^th^ percentile according to the reference population [26]. This indicates that, despite the lower rKVs in LBW individuals, oligonephropathy is not more prevalent in this population. Sanderson et al. [33] found no significant difference in rKV in their cohort of ELBW adolescents compared to the same reference population [26]. However, their cohort consisted of only 42 children which likely contributed to the lack of statistical significance. In the same study, they reported that 14.3% of children had rKV below the 10^th^ percentile. The higher percentage compared to our study could be explained by the lower mean BW in their cohort compared to ours (770.0 ± 173.1 g vs. 1109.7 ± 315.4 g).

Coffee consumption during pregnancy appeared to be a protective factor for both eGFR and KV. There are studies suggesting the protective effect of caffeine on neonatal AKI [34]. However, when interviewing parents, we were specifically interested in coffee consumption and therefore could have missed other beverages containing caffeine. Additionally, recall bias could have occurred as we inquired about information from 5–6 years ago. For these reasons, our results are not conclusive. We suggest that a study designed specifically to investigate the impact of caffeine intake during pregnancy on offspring kidney outcomes should be conducted.

There were only three ultrasound abnormalities (one hydronephrosis, one nephrocalcinosis, one simple cyst), and altered echogenicity was found in five patients. In a previous study, nephrocalcinosis was prevalent in infants born with LBW with especially high prevalence in those born ELBW [35]. In our cohort, only one patient had nephrocalcinosis. Our study suggests potential spontaneous resolution of nephrocalcinosis during childhood without specific consequences for the population, although this is only our assumption, as we do not know the prevalence of nephrocalcinosis in early childhood in our cohort. From those patients with altered echogenicity, four were born with ELBW, one of them has already been published as a case report (the patient with BW of 370 g) [27]. We believe the altered echogenicity is the result of abnormal kidney development due to LBW, as we did not identify another explanation for this, such as cystic kidney disease or glomerulonephritis. Our hypothesis is supported by the altered echogenicity being found mainly in ELBW children who are expected to have the worst kidney outcomes.

We found an elevated U-ca/cr in 26.2% of our patients, which is similar to a previous report [36]. We also found a negative correlation between U-α1/cr and KV, and small correlation with eGFR almost reached significance. Matsumura et al. [36] described a high prevalence of tubular dysfunction (the definition based on urine abnormalities) in ELBW individuals. During follow-up, some parameters improved while others remained stable or even got worse [36]. Zaffanello et al. [18] found higher excretion of α1-microglobulin in ELBW compared to VLBW children. The real significance of these urine abnormalities for the patients is questionable. In our study, no patient had either any electrolyte, acid–base balance, urine sediment or biochemistry abnormality, and only one child was found to have nephrocalcinosis. As there was a negative correlation between U-α1/cr and KV, the U-α1/cr may be a possible indirect marker of a quality of kidney development, but KV by itself gives us a lot of information and is easily estimated by kidney ultrasound, which is a cheap and readily available examination. Further investigation will show whether urine tubular abnormalities in LBW individuals may have a real clinical impact on patients’ health and diagnostic approach.

The strength of our study is its complexity. We investigated the association between a significant number of exposures and various kidney parameters. Most of the exposures were obtained directly from hospital documentation, minimizing the risk for bias during collection. We used creatinine-CyC-based equation, which is considered to provide the best performance. Additionally, our cohort included a significant number of twins, allowing us to study two different populations within the same study protocol, and we provided data on twins, which are scarce.

Our study has some limitations. We had to separate our cohort into two smaller groups due to the high proportion of twins in our cohort. However, studies on twins bring valuable information about individuals with similar environments and genetic predispositions. Also, there might be a selection bias in our study. However, children in our cohort had very similar distributions of birth parameters compared to excluded patients; we therefore believe that our cohort represents the standard distribution in the population. We were only able to investigate correlations and associations within our cohort, as there was no control group in the study. This was a cross-sectional study which does not allow us to describe changes of investigated parameters during a time period. We studied the potential effect of coffee drinking during pregnancy; however, there was a high probability of confounding and recall bias. Therefore, even though our results were significant, they are inconclusive. We found that rKV of both kidneys were lower in our cohort compared to normative data. However, we used the kidney volumes from the whole cohort mixing singletons and twins together.

In conclusion, in our study on 5-year-old LBW children, we found many factors to be associated with eGFR and KV in this population. Some of these factors differed between twins and singletons, which shows how many variables may have a significant impact on kidney health in these individuals. Patients born with a BW ≥ 1000 g are still able to maintain normal eGFR, while eGFR in ELBW patients significantly decreases with decreased BW. Importantly, eGFR seems to be better predicted by KV than by BW and gestational age in LBW children based on the multivariable models. BW and growth parameters correlated with KV, and tKV is in general better associated with all investigated parameters than rKV. Relative kidney volumes were significantly lower for both kidneys compared to the reference population; however, we did not find a higher prevalence of oligonephropathy in our cohort. LBW individuals, especially those born ELBW, may have altered kidney echogenicity likely as a sign of abnormal kidney development associated with LBW. Tubular dysfunctions in LBW individuals have questionable impact in clinical practice, as no laboratory signs such as electrolyte, acid–base balance, urine sediment or biochemistry abnormalities were found in our study.

## Supplementary Information

Below is the link to the electronic supplementary material.

Graphical abstract (PPTX 81.1 KB)

Supplementary file2 (XLSX 106 KB)

Supplementary file3 (DOCX 18 KB)

Supplementary file4 (DOCX 26 KB)

## Declarations

### Data availability

Complete data is available in the article and its supplementary materials.
