# Supplementary material for: Kidney volume and function of low-birth-weight children at 5 years: impact of singleton and twin birth
Source: Pediatr Nephrol. 2024 Oct 25;40(3):773–85. doi: 10.1007/s00467-024-06554-8 (PMC11746971; doi:10.1007/s00467-024-06554-8)
Supplement: Supplementary file 1 — Graphical abstract (PPTX 81.1 KB) [file 467_2024_6554_MOESM1_ESM.pptx]

## Slide 1
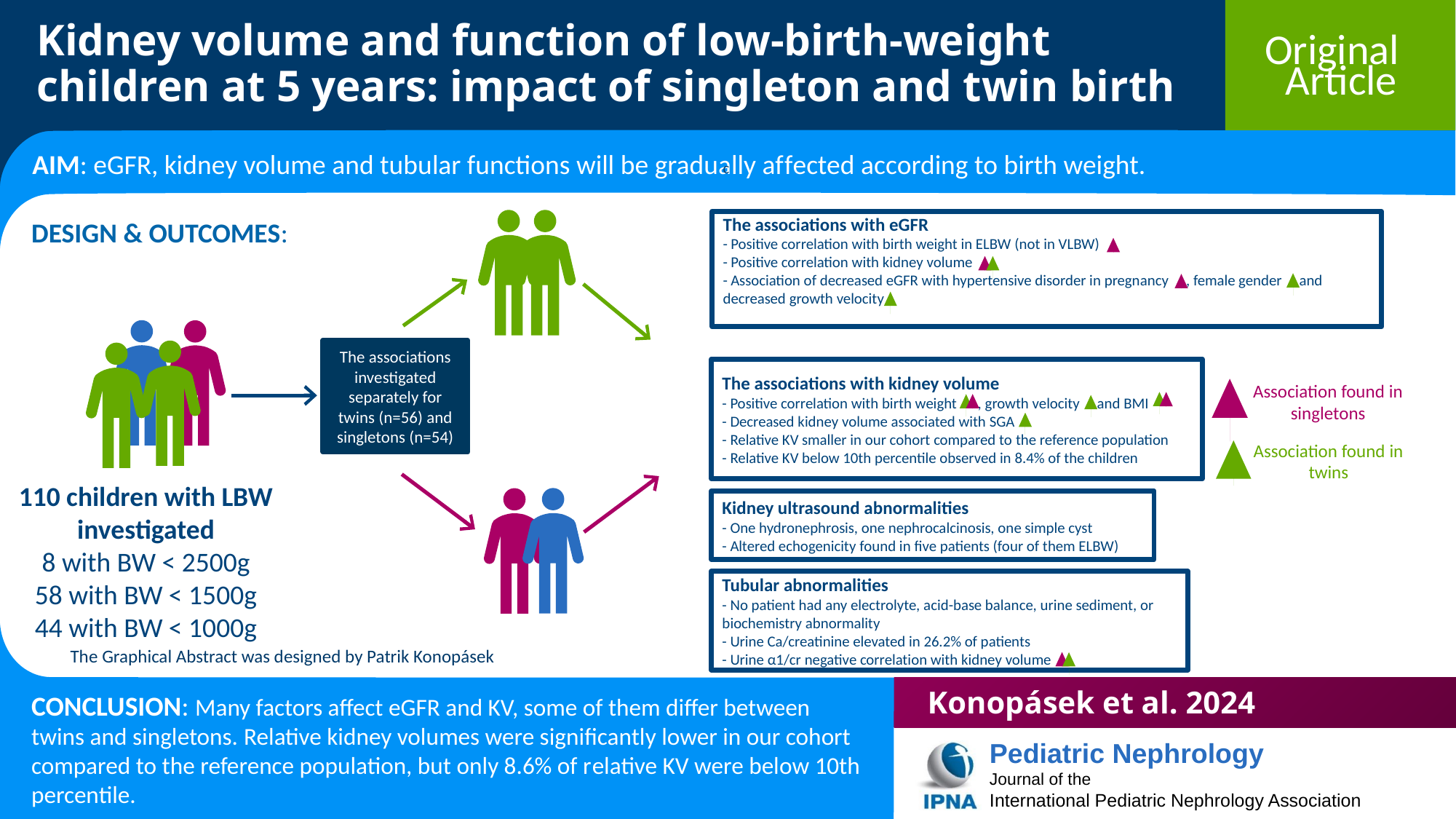

Kidney volume and function of low-birth-weight children at 5 years: impact of singleton and twin birth
AIM: eGFR, kidney volume and tubular functions will be gradually affected according to birth weight.
DESIGN & OUTCOMES:
c
The associations with eGFR
- Positive correlation with birth weight in ELBW (not in VLBW)
- Positive correlation with kidney volume
- Association of decreased eGFR with hypertensive disorder in pregnancy , female gender and decreased growth velocity
The associations investigated separately for twins (n=56) and singletons (n=54)
The associations with kidney volume
- Positive correlation with birth weight , growth velocity and BMI
- Decreased kidney volume associated with SGA
- Relative KV smaller in our cohort compared to the reference population
- Relative KV below 10th percentile observed in 8.4% of the children
Association found in singletons
Association found in twins
110 children with LBW investigated
8 with BW < 2500g
58 with BW < 1500g
44 with BW < 1000g
Kidney ultrasound abnormalities
- One hydronephrosis, one nephrocalcinosis, one simple cyst
- Altered echogenicity found in five patients (four of them ELBW)
Tubular abnormalities
- No patient had any electrolyte, acid-base balance, urine sediment, or biochemistry abnormality
- Urine Ca/creatinine elevated in 26.2% of patients
- Urine α1/cr negative correlation with kidney volume
The Graphical Abstract was designed by Patrik Konopásek
Konopásek et al. 2024
CONCLUSION: Many factors affect eGFR and KV, some of them differ between twins and singletons. Relative kidney volumes were significantly lower in our cohort compared to the reference population, but only 8.6% of relative KV were below 10th percentile.
