# Supplementary material for: Kidney volume and function of low-birth-weight children at 5 years: impact of singleton and twin birth
Source: Pediatr Nephrol. 2024 Oct 25;40(3):773–85. doi: 10.1007/s00467-024-06554-8 (PMC11746971; doi:10.1007/s00467-024-06554-8)
Supplement: Supplementary file 3 — Supplementary file3 (DOCX 18 KB) [file 467_2024_6554_MOESM3_ESM.docx]

Prenatal and perinatal characteristics and urine parameters of the cohort presented as mean ± standard deviations or total number + percentage

| *Parameter* | *Cohort (n=110)* | *Singletons (n=54)* | *Twins (n=56)* |
| --- | --- | --- | --- |
| Family history of hypertension | 32 (29.36%) | 17 (32.08%) | 15 (26.79%) |
| Coffee drinking during pregnancy | 35 (32.11%) | 23 (42.59%) | 12 (21.43%) |
| Maternal anemia | 20 (18.35%) | 8 (15.09%) | 12 (21.43%) |
| Smoking during pregnancy | 11 (10%) | 7 (13.21%) | 4 (7.14%) |
| Alcohol drinking during pregnancy | 2 (1.83%) | 1 (1.89%) | 1 (1.79%) |
| Gestational diabetes | 9 (8.26%) | 5 (9.43%) | 4 (7.14%) |
| Hypertensive disorder of pregnancy | 32 (29.09) | 20 (37.04%) | 12 (21.43%) |
| Cesarean section | 93 (84.55%) | 38 (70.37%) | 55 (98.21%) |
| Antenatal corticosteroids | 99 (90%) | 48 (88.89%) | 51 (91.07%) |
| Acute kidney injury | 7 (6.48%) | 5 (9.43%) | 2 (3.64%) |
| Aminoglycosides | 41 (37.61%) | 25 (47.17%) | 16 (28.57%) |
| Furosemide | 24 (22.02%) | 12 (22.64%) | 12 (21.43%) |
| Nonsteroidal analgesics | 7 (6.42%) | 2 (3.77%) | 5 (8.93%) |
| Patent ductus arteriosus | 20 (18.52%) | 10 (19.23%) | 10 (17.86%) |
| Bronchopulmonary dysplasia | 35 (35.71%) | 22 (50%) | 13 (24.07%) |
| Necrotizing enterocolitis | 5 (4.63%) | 1 (1.92%) | 4 (7.14%) |
| Sepsis | 17 (15.60%) | 9 (16.98%) | 8 (14.29%) |
| Urine α1/cr (µkat/mmol) | 0.78 ± 0.59 | 0.70 ± 0.57 | 0.85 ± 0.60 |
| Urine β2/cr (mg/mmol) | 0.024 ± 0.052 | 0.022 ± 0.030 | 0.028 ± 0.066 |
| Urine ca/cr (mmol/mmol) | 0.48 ± 0.38 | 0.48 ± 0.32 | 0.50 ± 0.42 |
| Urine ca/cr > 0.65 mmol/mmol | 28 (26.17%) | 13 (25.49%) | 15 (26.79%) |
