# Supplementary material for: Kidney volume and function of low-birth-weight children at 5 years: impact of singleton and twin birth
Source: Pediatr Nephrol. 2024 Oct 25;40(3):773–85. doi: 10.1007/s00467-024-06554-8 (PMC11746971; doi:10.1007/s00467-024-06554-8)
Supplement: Supplementary file 4 — Supplementary file4 (DOCX 26 KB) [file 467_2024_6554_MOESM4_ESM.docx]

Comparison of estimated glomerular filtration rate, total kidney volume and relative kidney volume across categorical variables in singletons and twins

| Comparison of estimated glomerular filtration rate across categorical variables in singletons | | | |
| --- | --- | --- | --- |
| Investigated parameter | Mean eGFR (factor not present vs present) in ml/min/1.73m^2^ | 95% CI | *P-*Value |
| Male gender | 88.16 vs 93.84 | -11.55 – 0.19 | 0.0577 |
| Birth weight < 1000g | 94.55 vs 88.72 | -0.26 – 11.92 | 0.0601 |
| Small for gestational age | 92.07 vs 87.70 | -5.15 – 13.87 | 0.3291 |
| Coffee drinking during pregnancy | 93.43 vs 87.72 | -0.56 – 11.99 | 0.0728 |
| Maternal anemia | 91.00 vs 90.67 | -5.68 – 6.34 | 0.9086 |
| *Hypertensive disorder of pregnancy* | *93.86 vs 87.26* | *0.73* – *12.48* | *0.0285** |
| Family history of hypertension | 91.76 vs 89.24 | -3.04 – 8.10 | 0.3655 |
| Cesarean section | 91.54 vs 91.37 | -6.47 – 6.81 | 0.9583 |
| Antenatal corticosteroids | 95.10 vs 90.25 | -1.72 – 11.41 | 0.1410 |
| Acute kidney injury | 92.78 vs 82.41 | -2.06 – 22.78 | 0.0846 |
| Aminoglycosides use | 91.75 vs 91.85 | -6.20 – 6.00 | 0.9736 |
| Furosemide use | 93.11 vs 87.34 | -0.74 – 12.28 | 0.0796 |
| Patent ductus arteriosus | 92.40 vs 89.93 | -7.17 – 12.11 | 0.5872 |
| Bronchopulmonary dysplasia | 93.44 vs 90.28 | -3.50 – 9.81 | 0.3433 |
| Sepsis | 92.17 vs 90.76 | -6.16 – 8.99 | 0.7010 |
| Comparison of estimated glomerular filtration rate across categorical variables in twins | | | |
| Investigated parameter | Mean eGFR (factor not present vs present) in ml/min/1.73m^2^ | 95% CI | *P-*Value |
| Male gender | 89.88 vs 94.58 | -14.51 – 0.16 | 0.0553 |
| Birth weight < 1000g | 92.16 vs 90.52 | -4.95 – 9.45 | 0.5401 |
| Small for gestational age | 92.75 vs 86.35 | -0.89 – 11.54 | 0.0930 |
| *Coffee drinking during pregnancy* | *89.21 vs 100.94* | *-16.55* – *(-7.43)* | *< 0.0001** |
| Maternal anemia | 91.48 vs 92.61 | -10.01 – 5.73 | 0.5945 |
| Hypertensive disorder of pregnancy | 90.86 vs 94.84 | -10.98 – 4.00 | 0.3613 |
| Family history of hypertension | 91.81 vs 91.46 | -6.47 – 6.99 | 0.9392 |
| Antenatal corticosteroids | 96.35 vs 89.35 | -0.47 – 12.90 | 0.0686 |
| Aminoglycosides use | 91.25 vs 92.90 | -11.39 – 7.10 | 0.6500 |
| Furosemide use | 91.94 vs 90.95 | -5.32 – 9.32 | 0.5918 |
| Nonsteroidal analgesics use | 91.72 vs 91.73 | -6.88 – 11.16 | 0.6422 |
| Patent ductus arteriosus | 89.91 vs 100.07 | -17.63 – 0.65 | 0.0686 |
| Bronchopulmonary dysplasia | 90.74 vs 94.44 | -10.37 – 6.73 | 0.6760 |
| Sepsis | 91.23 vs 94.69 | -10.43 – 4.05 | 0.3876 |
| Comparison of total kidney volume across categorical variables in singletons | | | |
| Investigated parameter | Mean total kidney volume (factor not present vs present) in ml | 95% CI | *P-*Value |
| Male gender | 85.28 vs 89.01 | -12.37 – 4.91 | 0.3889 |
| *Birth weight < 1000g* | *92.54 vs 82.83* | *1.64 – 17.80* | *0.0194** |
| Small for gestational age | 88.71 vs 79.14 | -4.95– 24.10 | 0.1664 |
| Coffee drinking during pregnancy | 89.95 vs 83.24 | -1.79 – 15.21 | 0.1188 |
| Maternal anemia | 87.12 vs 86.24 | -6.65 – 8.40 | 0.8093 |
| Hypertensive disorder of pregnancy | 90.26 vs 82.15 | -0.31 – 16.54 | 0.0585 |
| Family history of hypertension | 87.01 vs 86.98 | -8.54 – 8.60 | 0.9945 |
| Cesarean section | 90.39 vs 86.03 | -5.68 – 14.41 | 0.3791 |
| Antenatal corticosteroids | 89.27 vs 86.76 | -4.55 – 9.58 | 0.4770 |
| Acute kidney injury | 88.58 vs 84.09 | -27.69 – 36.65 | 0.6969 |
| Aminoglycosides use | 86.33 vs 90.26 | -11.97 – 4.12 | 0.3307 |
| Furosemide use | 89.23 vs 84.64 | -5.47 – 14.65 | 0.3483 |
| Patent ductus arteriosus | 88.92 vs 84.70 | -7.13 – 15.57 | 0.4365 |
| Bronchopulmonary dysplasia | 90.86 vs 86.22 | -4.24 – 13.51 | 0.2966 |
| Sepsis | 87.87 vs 89.20 | -12.96 – 10.30 | 0.8116 |
| Comparison of relative kidney volume across categorical variables in singletons | | | |
| Investigated parameter | Mean relative kidney volume (factor not present vs present) in ml/m^2^ | 95% CI | *P-*Value |
| Male gender | 122.12 vs 127.58 | -21.11 – 10.19 | 0.4853 |
| Birth weight < 1000g | 131.05 vs 120.05 | -4.37 – 26.38 | 0.1560 |
| Small for gestational age | 126.80 vs 115.37 | -5.36 – 28.21 | 0.1637 |
| Coffee drinking during pregnancy | 125.99 vs 121.79 | -10.13 – 18.54 | 0.5579 |
| Maternal anemia | 124.32 vs 123.06 | -15.65 – 18.17 | 0.8736 |
| Hypertensive disorder of pregnancy | 128.79 vs 118.70 | -3.20 – 23.37 | 0.1335 |
| Family history of hypertension | 123.74 vs 124.94 | -16.39 – 14.01 | 0.8751 |
| Cesarean section | 128.33 vs 123.81 | -15.01 – 24.05 | 0.6355 |
| Antenatal corticosteroids | 122.50 vs 126.16 | -19.03 – 11.71 | 0.6293 |
| Acute kidney injury | 126.66 vs 119.33 | -32.38 – 47.03 | 0.6226 |
| Aminoglycosides use | 123.50 vs 128.86 | -20.77 – 10.03 | 0.4857 |
| Furosemide use | 128.92 vs 115.96 | -1.42 – 27.35 | 0.0753 |
| Patent ductus arteriosus | 126.80 vs 125.07 | -19.83 – 23.29 | 0.8651 |
| Bronchopulmonary dysplasia | 126.53 vs 126.40 | -16.48 – 16.75 | 0.9868 |
| Sepsis | 123.09 vs 134.57 | -31.24 – 8.28 | 0.2379 |
| Comparison of total kidney volume across categorical variables in twins | | | |
| Investigated parameter | Mean total kidney volume (factor not present vs present) in ml | 95% CI | *P-*Value |
| Male gender | 85.27 vs 88.39 | -12.87 – 7.96 | 0.6441 |
| Birth weight < 1000g | 87.13 vs 84.11 | -1.24 – 17.22 | 0.0898 |
| *Small for gestational age* | *89.20 vs 70.88* | *11.78 – 22.89* | *< 0.0001** |
| *Coffee drinking during pregnancy* | *82.69 vs 99.76* | *-31.01 – (-4.53)* | *0.0085** |
| Maternal anemia | 87.47 vs 83.04 | -8.95 – 18.27 | 0.4512 |
| Hypertensive disorder of pregnancy | 84.88 vs 92.77 | -23.29 – 9.47 | 0.4084 |
| Family history of hypertension | 86.44 vs 86.64 | -9.22 – 10.76 | 0.8801 |
| Antenatal corticosteroids | 90.61 vs 84.43 | -3.10 – 17.00 | 0.1751 |
| Aminoglycosides use | 86.07 vs 87.57 | -15.29 – 10.89 | 0.7415 |
| Furosemide use | 86.32 vs 87.07 | -8.20 – 14.53 | 0.5851 |
| Nonsteroidal analgesics use | 86.23 vs 89.75 | -17.97 – 18.96 | 0.9581 |
| Patent ductus arteriosus | 85.93 vs 89.25 | -13.19 – 9.45 | 0.7460 |
| Bronchopulmonary dysplasia | 85.62 vs 88.68 | -9.90 – 16.41 | 0.6277 |
| Sepsis | 86.23 vs 87.96 | -6.78 – 12.17 | 0.5773 |
| Comparison of relative kidney volume across categorical variables in twins | | | |
| Investigated parameter | Mean relative kidney volume (factor not present vs present) in ml/m^2^ | 95% CI | *P-*Value |
| Male gender | 122.01 vs 123.90 | -17.80 – 7.83 | 0.4457 |
| Birth weight < 1000g | 124.01 vs 119.13 | -0.29 – 23.22 | 0.0559 |
| *Small for gestational age* | 125.23 vs 108.47 | 12.30 – 25.82 | < 0.0001* |
| *Coffee drinking during pregnancy* | 118.61 vs 137.24 | -36.00 – (-3.84) | 0.0152* |
| Maternal anemia | 122.51 vs 123.58 | -16.94 – 15.48 | 0.9296 |
| Hypertensive disorder of pregnancy | 119.81 vs 134.23 | -33.43 – 6.51 | 0.1864 |
| Family history of hypertension | 122.95 vs 122.10 | -11.23 – 20.08 | 0.5794 |
| Antenatal corticosteroids | 129.48 vs 119.38 | -4.75 – 24.45 | 0.1861 |
| Aminoglycosides use | 122.38 vs 123.70 | -17.25 – 13.08 | 0.7878 |
| Furosemide use | 124.02 vs 118.31 | -0.80 – 19.61 | 0.0709 |
| Nonsteroidal analgesics use | 122.77 vs 122.49 | -14.92 – 11.72 | 0.8136 |
| Patent ductus arteriosus | 122.35 vs 124.72 | -15.28 – 6.57 | 0.4345 |
| Bronchopulmonary dysplasia | 121.56 vs 123.22 | -10.70 – 14.57 | 0.7644 |
| Sepsis | 123.13 vs 120.57 | -1.63 – 14.99 | 0.1151 |
